# ‘*Caribbean Creep*’ Chills Out: Climate Change and Marine Invasive Species

**DOI:** 10.1371/journal.pone.0029657

**Published:** 2011-12-28

**Authors:** João Canning-Clode, Amy E. Fowler, James E. Byers, James T. Carlton, Gregory M. Ruiz

**Affiliations:** 1 Smithsonian Environmental Research Center, Edgewater, Maryland, United States of America; 2 IMAR / Department of Oceanography and Fisheries, University of the Azores, Horta, Portugal; 3 Center of Oceanography, Faculty of Sciences, University of Lisbon, Lisbon, Portugal; 4 The University of Georgia, Athens, Georgia, United States of America; 5 Williams College – Mystic Seaport, Mystic, Connecticut, United States of America; University of Hamburg, Germany

## Abstract

**Background:**

New marine invasions have been recorded in increasing numbers along the world's coasts due in part to the warming of the oceans and the ability of many invasive marine species to tolerate a broader thermal range than native species. Several marine invertebrate species have invaded the U.S. southern and mid-Atlantic coast from the Caribbean and this poleward range expansion has been termed ‘*Caribbean Creep*’. While models have predicted the continued decline of global biodiversity over the next 100 years due to global climate change, few studies have examined the episodic impacts of prolonged cold events that could impact species range expansions.

**Methodology/Principal Findings:**

A pronounced cold spell occurred in January 2010 in the U.S. southern and mid-Atlantic coast and resulted in the mortality of several terrestrial and marine species. To experimentally test whether cold-water temperatures may have caused the disappearance of one species of the ‘Caribbean Creep’ we exposed the non-native crab *Petrolisthes armatus* to different thermal treatments that mimicked abnormal and severe winter temperatures. Our findings indicate that *Petrolisthes armatus* cannot tolerate prolonged and extreme cold temperatures (4–6°C) and suggest that aperiodic cold winters may be a critical ‘reset’ mechanism that will limit the range expansion of other ‘Caribbean Creep’ species.

**Conclusions/Significance:**

We suggest that temperature ‘aberrations’ such as ‘cold snaps’ are an important and overlooked part of climate change. These climate fluctuations should be accounted for in future studies and models, particularly with reference to introduced subtropical and tropical species and predictions of both rates of invasion and rates of unidirectional geographic expansion.

## Introduction

Invasive species and climate change are among the most critical pressures to biodiversity [1], [2]. Despite contributing to shifts in the distribution of native species, climate change also facilitates the establishment and the range extension of invasive species [1], [3]. Several studies have determined that certain invasive species have a superior capacity to tolerate increased temperatures (as compared to native species) (e.g. [4], [5], [6]). This broader thermal repertoire may be a key aspect in the rising number of new invasions in recent years. When investigator bias (increased interest in invasions leading to increased number of invasions) is removed, this trend still persists [7]. Several marine invertebrate species, such as the barnacle *Megabalanus coccopoma*, the marsh snail *Creedonia succinea*, the green mussel *Perna viridis*, and the porcelain crab *Petrolisthes armatus*, have invaded the U.S. southern and mid-Atlantic coast from the Caribbean and other southern waters [8]. This poleward range expansion has been termed the ‘Caribbean Creep’, but the general phenomenon is not restricted to the US Atlantic coast and has been observed along other coasts worldwide [9].

**Figure 1:**
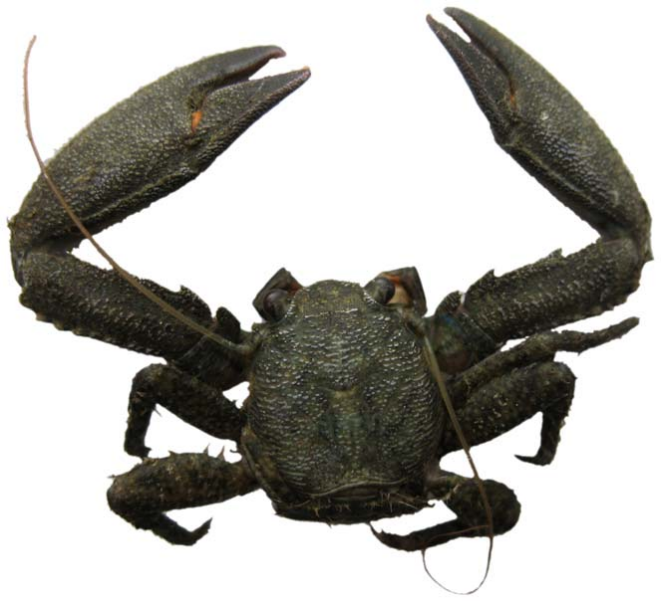
The green porcelain crab *Petrolisthes armatus* can be found in rock rubble or oyster reefs in shallow intertidal or subtidal areas. It occurs in the western Atlantic (Gulf of Mexico, Bermuda, the West Indies and Caribbean, and South America to southern Brazil), eastern Atlantic (tropical western Africa and Ascension Island), and eastern Pacific (Gulf of California to Peru). Since the 1990s it has invaded the Mid-Atlantic (South Carolina to Florida).

Various models have predicted the continued decline of global biodiversity over the next 100 years due to global climate change [4], [10], but these have focused predominantly on mean temperature increase, despite the likely importance of periodic climate fluctuations such as heat and cold waves [11]. While many studies consider future effects of global warming on altered distributions of species, few evaluate the episodic impacts of prolonged cold events that could impact range expansion. Additionally, several studies have discussed winter mass mortalities of intertidal and shallow-water marine invertebrates (see e.g. [12], [13], [14], [15]) as well as mechanistic impacts of climate change in fish populations [16], but not as linked to range expansion dynamics under climate change models. The potential significance of such events is illustrated by a pronounced cold spell during January 2010 along the southeastern U.S. coast resulting in the mortality of native and non-native species such as manatees, sea turtles, crocodiles, iguanas, pythons, fish and corals (see e.g. [17], [18]). These abnormal cold temperatures experienced in southeastern U.S. coast during the period of 28 December 2009 to 13 January 2010 are associated with extremely negative values of the North Atlantic Oscillation (NAO) index [19]. Several ‘Caribbean Creep’ species underwent dramatic declines surrounding this event. For example, the non-native green mussel *Perna viridis*, suffered mass mortalities in Tampa Bay, Florida as mussels were present during field surveys in October 2008 and 2009 and not found after the cold spell [20]. To experimentally test whether cold-water temperatures may have caused the disappearance of other ‘Caribbean Creep’ species, we exposed the porcelain crab *Petrolisthes armatus* (Fig. 1) to different thermal treatments that mimicked severe winter temperatures. Using data from the National Data Buoy Center for Fort Pulanski, Georgia, we replicated the temperatures for the 25 days of severe temperature fluctuation in January 2010.

## Materials and Methods

### Animal collection

Adult *Petrolisthes armatus* (carapace width 6–11mm) were hand collected from the Wilmington River, near the mouth of the Wassaw Sound, in Savannah, Georgia on November 17, 2010, to coincide with the onset of winter low temperatures, and transported to the Smithsonian Environmental Research Center in Edgewater, MD, USA. At the laboratory, the crabs were acclimatized for 10 days in three aquaria containing 20 L of 0.5 µm filtered seawater adjusted to 32±1‰ (hereafter FSW) and 15±1°C, mimicking current natural conditions of the field site. The crabs were fed pellet food ad libidum, and any uneaten food was removed during full exchange of fresh sea water (FSW) every other day. The light:dark cycle was 12∶12 h during both the acclimatization and experimental periods. No specific permits were required for the described field studies. Collection sites were not privately owned or protected and field studies did not involve endangered or protected species.

### Field survey

In August 2010, near the same site where crabs were collected for lab trials and the same area where reference [21] measured crab densities were taken, we randomly placed a 0.5m×0.5m quadrat on 5 replicate oyster reefs which were separated by a minimum of 100m. Within each quadrat, we quickly excavated all oysters, organisms, and associated mud on the surface and placed material into a plastic tote with lid. At the lab we placed tote contents into a large sieve with 1mm steel mesh and thoroughly sieved and sorted all material, counting and collecting *Petrolisthes armatus.*

### Experimental design

**Figure 2:**
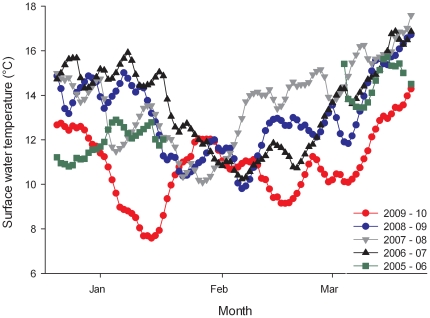
Water surface temperatures (°C) from winter 2005–06 until winter 2009–10 at Fort Pulaski, Georgia (12 km from crab collection site). Average daily temperatures (n = 24) from the National Data Buoy Center are indicated.

**Figure 3:**
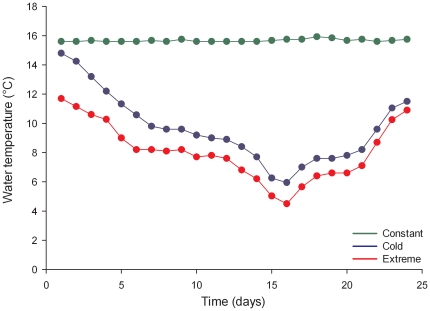
We exposed the invasive green porcelain crab *Petrolisthes armatus* to three experimental temperature ranges: constant 15°C (green); cold based on January 2010 temperatures: 5.3–14.4°C (blue); extreme: 4.1–11.7°C (red) for 25 days. Water temperatures from the three treatments extracted with data loggers are shown.

Acclimated *P. armatus* were randomly placed into separate plastic 50mL containers (one crab per container) of FSW calibrated to 15±1°C, and a lid loosely secured. Crabs and containers were then placed in one of three incubators, and the temperature manually calibrated to concurrently mimic historical observed fluctuations in water temperature collected from the National Data Buoy Center for Fort Pulanski, Georgia (12 km from crab collection site). We replicated the temperatures for the 25 days of severe temperature fluctuation in January 2010 (Fig. 2). Our treatments contained 18 crabs and simulated three different scenarios: i) constant (15.5±0.1°C, in green in Fig. 3); ii) cold treatment (5.3–14.4°C, in blue) that mimicked January 2010 water temperature oscillations, and iii) extreme treatment (4.1–1.7°C, in red) to simulate an even more severe winter (Fig. 3). For each treatment, the 18 crabs were placed in individual containers with no water exchange. Temperature was manipulated by +/−1°C every 24 h over a period of 24 days (November 30– December 24, 2010). Temperature data loggers were submerged in independent containers and stationed in each incubator. Crabs were exposed to the treatment temperature for 24 h, after which survival was assessed based on the presence of movement (either autonomous or when probed). Crabs were fed pellet food ad libidum every other day, and FSW, previously calibrated to the treatment temperature, was exchanged daily. Survival rates between different treatments were compared with the Log-rank test.

## Results and Discussion

**Figure 4:**
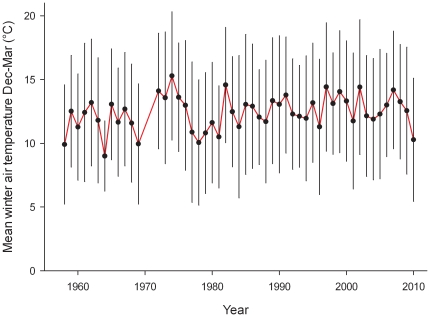
Historical winter (120 days from December to March; n = 120) air temperature (°C) for 1958–2006 was collected at the University of Georgia Marine Institute on Sapelo Island, Georgia (65 km south of crab collection site). Data from 2007–2010 are from the USGS Climate Station at Hudson Creek, Georgia (65 km south of collection site). Error bars represent the standard deviation of the mean.

**Figure 5:**
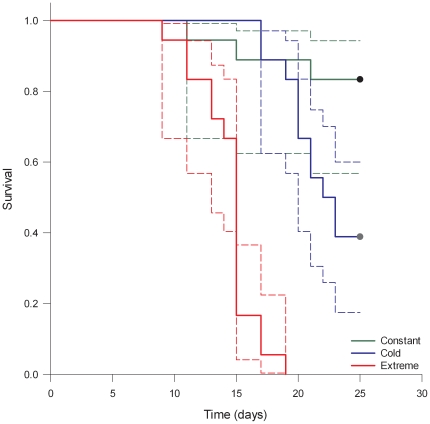
Kaplan-Meyer survival curves of *Petrolisthes armatus* collected in Georgia and exposed to three thermal treatments. Color codes as in Fig. 3. Dashed lines indicate 95% confidence intervals.

Following the trend of other ‘Caribbean Creep’ species (e.g. [20]), the non-native porcelain crab *Petrolisthes armatus* also declined from 2007 summer densities [21] that ranged from 790–11,200 crabs/m^2^ to 2010 summer densities of 0–12/m^2^ at the same sites in Georgia, where the coast experienced the lowest winter temperatures since 1978 and the third lowest since 1958 (Fig. 4). Although Hollebone and Hay employed a different methodology for the field survey (basket traps *versus* quadrat techniques) the decrease in densities of *Petrolisthes* by several orders of magnitude cannot be exclusively attributed to different counting techniques. The two colder treatments caused elevated mortality of *Petrolisthes*, manifested after 9 days in the extreme treatment and after 17 days in the cold treatment. At the end of the experiment, 83% of crabs survived in the constant treatment, 39% survived the cold temperatures that mimicked the January 2010 episode, while no crabs survived the extreme treatment (Fig. 5). There was a statistically significant difference between survival curves (Log-rank test: χ^2^ = 53.5, p<0.001). Range expansions are not exclusively triggered by temperature as other factors such as human mediated propagule supply, habitat destruction, and species interactions may also affect range shifts and boundaries. In addition, it is well known that weather extremes may reshape and forge the range of marine organisms (see e.g. [22], [23], [24], [25], [26]). However, that such extremes apply to resetting (either by slowing or accelerating) predictions of range advances with a warming world appears not to have been previously considered.

While these experimental results suggest that the single, short-duration cold snap would not by itself diminish the crab populations to the post-winter levels seen in the field, reduced temperatures may in fact significantly compound crab mortality in secondary and tertiary ways. For example, we observed that cold temperatures decreased the crab's mobility, which in natural settings may increase susceptibility to predation and/or influence feeding behavior. Also, prolonged exposure to cold temperatures may further compromise the crab's physiological ability to overcome the cumulative effect of multiple cold events. Although our experiment only mimicked the first temperature dip of the 2010 cold spell, this event was followed by two more severe cold fluctuations in February and March (Fig. 2). If each cold event had the same impact of decreasing the population by 60% each time, these cumulative episodes could explain the observed pattern of decline in *Petrolisthes* populations in Georgia, USA.

These results indicate that *Petrolisthes armatus* cannot tolerate prolonged and extreme cold temperatures (4–6°C) and suggest that aperiodic cold winters may be a critical ‘reset’ mechanism, serving to reduce local abundance and limit the range expansion rates of ‘Caribbean Creep’ species. Recent projections anticipate that marine species from the U.S. southern and mid-Atlantic coast will shift northward due to climate change at a rate higher than 2 km per year [10]. However, temperature ‘aberrations’ such as ‘cold snaps’ are also an important, and often overlooked, part of global climate change. Global climate patterns clearly affect species range expansions, both by advancing and hindering their migration up coastlines. We emphasize that these episodic climate fluctuations should be accounted for in future studies, particularly with reference to introduced tropical species and attempts to predict both rates of invasion and rates of unidirectional geographic expansion.
